# The bifurcation angle is associated with the progression of saccular aneurysms

**DOI:** 10.1038/s41598-022-11160-5

**Published:** 2022-05-06

**Authors:** Kampei Shimizu, Hiroharu Kataoka, Hirohiko Imai, Takeshi Miyata, Akihiro Okada, Nobuyuki Sakai, Masaki Chin, Koichi Iwasaki, Taketo Hatano, Hirotoshi Imamura, Ryota Ishibashi, Masanori Goto, Masaomi Koyanagi, Tomohiro Aoki, Susumu Miyamoto

**Affiliations:** 1 Department of Neurosurgery, Kyoto University Graduate School of Medicine, Kyoto, 606-8507 Japan; 2 Department of Molecular Pharmacology, Research Institute, National Cerebral and Cardiovascular Center, Suita, 564-8565 Japan; 3 Department of Neurosurgery, National Cerebral and Cardiovascular Center, 6-1 Kishibe-Shimmachi, Suita City, Osaka 564-8565 Japan; 4 Department of Systems Science, Graduate School of Informatics, Kyoto University, Kyoto, 606-8507 Japan; 5 Department of Neurosurgery, Kobe City Medical Center General Hospital, Kobe, 650-0047 Japan; 6 Department of Neurosurgery, Kurashiki Central Hospital, Kurashiki, 710-8602 Japan; 7 Department of Neurosurgery, Tazuke Kofukai Medical Research Institute, Kitano Hospital, Osaka, 530-8480 Japan; 8 Department of Neurosurgery, Kokura Memorial Hospital, Kokura, 802-8555 Japan

**Keywords:** Aneurysm, Cerebrovascular disorders

## Abstract

The role of the bifurcation angle in progression of saccular intracranial aneurysms (sIAs) has been undetermined. We, therefore, assessed the association of bifurcation angles with aneurysm progression using a bifurcation-type aneurysm model in rats and anterior communicating artery aneurysms in a multicenter case–control study. Aneurysm progression was defined as growth by ≥ 1 mm or rupture during observation, and controls as progression-free for 30 days in rats and ≥ 36 months in humans. In the rat model, baseline bifurcation angles were significantly wider in progressive aneurysms than in stable ones. In the case–control study, 27 and 65 patients were enrolled in the progression and control groups. Inter-observer agreement for the presence or absence of the growth was excellent (κ coefficient, 0.82; 95% CI, 0.61–1.0). Multivariate logistic regression analysis showed that wider baseline bifurcation angles were significantly associated with subsequent progressions. The odds ratio for the progression of the second (145°–179°) or third (180°–274°) tertiles compared to the first tertile (46°–143°) were 5.5 (95% CI, 1.3–35). Besides, the bifurcation angle was positively correlated with the size of aneurysms (Spearman’s rho, 0.39; *P* = 0.00014). The present study suggests the usefulness of the bifurcation angle for predicting the progression of sIAs.

## Introduction

Saccular intracranial aneurysms (sIAs) develop preferentially at arterial bifurcations. The pathogenesis of sIAs has been considered to be mediated by mechanosensing of hemodynamic stress loaded there^1^. Meanwhile, the arterial bifurcation angle is an essential element for effectively maintaining homeostasis of the cardiovascular system; to minimize the cardiac workload required to drive the circulation, bifurcation angles have been hypothesized to be optimized following the principle of minimum work^2–4^. Several in vivo experiments have shown that bifurcation angles were consistent with the theoretical values determined by the radii and the blood flow of the involved arteries^5,6^. These findings imply that the arteries sense hemodynamic force to optimize the bifurcation angle. Moreover, past human studies analyzing the morphology of bifurcations have demonstrated that the angles formed by two daughter branches were larger in cases with sIAs than ones without sIAs^7–12^, suggesting that failure of this mechanosensing may provoke hemodynamic stress-dependent biological responses leading to the pathogenesis of sIAs.

Clinically, sIAs have been surgically or conservatively treated on a case-by-case basis, considering the estimated rupture risks and risks posed by surgical interventions^13^. During the past two decades, several factors, such as the size and location of lesions, have been used to estimate rupture risks^14^. In addition, the growth of sIAs has been suggested to be an important event predisposing to rupture^15,16^. The above-mentioned background indicates that the bifurcation angle may be a novel factor predicting the progression (i.e., growth or rupture) of sIAs. While past cross-sectional studies have shown that the presence of sIAs was associated with wider bifurcation angles^8,9,12,17^, a longitudinal study evaluating an association between the bifurcation angle and the progression has yet to be performed.

Therefore, we evaluated whether bifurcation angles are associated with progression defined as the growth or rupture of saccular aneurysms in rats and humans. We first investigated the potential contribution of bifurcation angles to the progression of saccular aneurysms using a rat bifurcation-type aneurysm model. Then, we further evaluated the clinical relevance of the findings acquired from the rat model in a case–control study. We selected anterior communicating artery (Acom) aneurysms as the study targets because of their high prevalence and high rupture risk per year compared to aneurysms at other locations^18,19^.

## Methods

The animal experiments complied with the National Institute of Health’s Guide for the Care and Use of Laboratory Animals and the Animal Research Reporting In Vivo Experiments (ARRIVE) guidelines. The Institutional Animal Care and Use Committee of the National Cerebral and Cardiovascular Center approved the protocol (Approval Number #19036 and #20003). The protocol of the human study was approved by Kyoto University Graduate School and Faculty of Medicine Ethics Committee, The Kobe City Medical Center General Hospital Research Ethics Committee, Kurashiki Central Hospital Medical Ethics Committee, Medical Ethics Committee of Kitano Hospital, and Kokura Memorial Hospital Clinical Research Ethics Committee (Approval Number #R1682, #zn190206, #3115, #P190100700, and #18121902). Informed consent from individual patients was waived by Kyoto University Graduate School and Faculty of Medicine Ethics Committee, The Kobe City Medical Center General Hospital Research Ethics Committee, Kurashiki Central Hospital Medical Ethics Committee, Medical Ethics Committee of Kitano Hospital, and Kokura Memorial Hospital Clinical Research Ethics Committee because of the minimal risk posed by the present study. Alternatively, opt-out enrolment of patients was conducted. The study was performed in accordance with the Declaration of Helsinki and the Strengthening the Reporting of Observational Studies in Epidemiology (STROBE) statement^20^. The datasets analyzed in the present study are available from the corresponding author on reasonable request.

### Rat aneurysm model

Seven-week-old, male, Sprague-Dawley rats were obtained from Japan SLC (Shizuoka, Japan). Rats were maintained on a light/dark cycle of 12 h/12 h and had free access to diet and water. Saccular aneurysms similar in morphology and histology to human sIAs were induced at surgically-created common carotid artery (CCA) bifurcations, as previously demonstrated^21,22^. Briefly, under general anesthesia using a combination of intraperitoneal injection of pentobarbital sodium (50 mg/kg) and inhalation of isoflurane (1.5–2.0%), the left CCA was anastomosed end-to-side to the right CCA with a 10-0 nylon suture. Hypervolemia was induced by a high-salt diet and ligation of the left renal artery. After the surgical manipulation, rats were fed a special diet containing 8% sodium chloride and 0.12% 3-aminopropionitrile (Tokyo Chemical Industry, Tokyo, Japan), an inhibitor of lysyl oxidase that catalyzes the cross-linking of collagen and elastin. Blood pressure was measured by the tail-cuff method. The experimental animals were checked once a day after aneurysm induction. Because saccular aneurysms in this model are asymptomatic and rupture of the lesions causes sudden death, the animals were not given painkillers during the observation period. They were sacrificed by an intraperitoneal injection of pentobarbital sodium (200 mg/kg) when their body weight decreased to less than 80% of their littermates, they could not take their diet, or behaved restlessly. Any animals that died during the observation period were autopsied to determine the cause of death, especially rupture of induced aneurysms.

### MR examination in rats

Morphological and blood flow data were acquired by magnetic resonance imaging (MRI) as described previously^21^. Briefly, MRI was conducted with a 7-Tesla preclinical scanner (BioSpec 70/20 USR; Bruker BioSpin MRI GmbH, Ettlingen, Germany) and a quadrature transmit-receive volume coil to detect MR signals (inner diameter 72 mm, T9562; Bruker BioSpin). During MRI, rats were placed under general anesthesia with inhalation of 3% isoflurane in air at 1.4 L/min through a face mask. Morphology of the CCAs was assessed by three-dimensional time-of-flight MR angiography. Blood flow volume at the right CCA proximal to the bifurcation during a cardiac cycle was estimated by cardiac-gated, two-dimensional, phase-contrast MRI. The acquisition parameters for the three-dimensional time-of-flight MR angiography and the phase-contrast MRI were the same as those in the previous study^21^.

MRI examination on the 30th day postoperatively was planned for all the rats. In a subgroup of rats, sequential MRI examinations on the 5th, 10th, 17th, and 30th days after the surgical manipulations were planned to detect the time course of morphological changes at the site of anastomosis. Morphological data of the CCA and induced aneurysms were visualized using three-dimensional volume rendering on Horos visualization software (64-bit, version 3.6.6, https://horosproject.org). The largest dimension of induced aneurysms and the bifurcation angle were measured.

### Design of the human study

Clinical and radiological data were retrospectively collected from the five large-volume centers in western Japan. A case–control study was performed to investigate whether bifurcation angles are associated with the progression of Acom aneurysms. Consecutive patients who visited each institutions during the study period were assessed for eligibility. Inclusion criteria was patients with an Acom aneurysm. Fusiform, mycotic, and dissecting aneurysms were excluded. Patients whose radiological data at the time of diagnosis were unavailable, poor in quality, or the Acom could not be identified were also excluded. Patients were categorized into the progression group if there was growth or rupture during follow-up. Growth of aneurysms was defined as an increase of the largest dimension by ≥ 1 mm. Patients with Acom aneurysms that were progression-free for ≥ 36 months from diagnosis were included in the control group. Assessments of the growth were independently performed by two investigators (i.e., one investigator was K.S. and the other was T.M. at Kyoto University Hospital, H.I. at Kobe City Medical Center General Hospital, R.I. at Kurashiki Central Hospital, M.G. at Tazuke Kofukai Medical Research Institute and Kitano Hospital, or M.K. at Kokura Memorial Hospital). Discrepancies of the assessments were resolved after discussion.

### Acquisition of clinical and radiologic data

Medical records and radiological data were reviewed to identify patients with Acom aneurysms showing progression between June 2006 and April 2019. To enroll control cases, consecutive outpatients with Acom aneurysms who visited the hospitals for a follow-up radiological examination were enrolled, and their medical records and radiological data at the time of diagnosis were reviewed between June 2006 and April 2019. Patients’ characteristics included age, sex, smoking, and history of hypertension, dyslipidemia, and diabetes mellitus. The radiological characteristics analyzed were the largest dimension and neck size of aneurysms, vessel diameters (the ipsilateral A1 and A2 segments, the Acom, and the contralateral A1 segment), angles formed between the Acom and A2 (Acom/A2 angle), and angles formed between the A1 and the plane including the proximal Acom and A2 (A1/Acom-A2 plane angle). Radiological data of three-dimensional rotational angiography, computed tomography angiography, or MR angiography were acquired in DICOM format. In the assessment of aneurysm growth, the size of aneurysms was compared between the same modalities.

**Figure 1:**
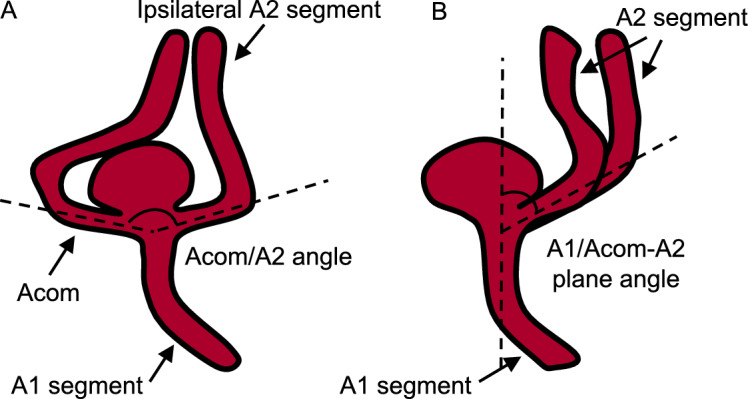
Schematic diagrams showing angle measurement at the anterior communicating artery (Acom)-anterior cerebral artery (ACA) bifurcation. The angles formed between the Acom and the A2 segment (the Acom/A2 angle) (**A**) and between the A1 segment and the plane containing both the Acom and the A2 segment (the A1/Acom-A2 plane angle) (**B**) are presented.

The diameter and angle of arteries were measured in a method similar to previous studies^8,10^. The diameters of the A1 segment and the Acom were measured at the midpoints of the segments. The diameter of the A2 segment was measured 5 mm beyond the Acom/A2 bifurcation apex. The Acom/A2 and A1/Acom-A2 plane angles were defined by setting three lines through the center axis of the distal A1 segment, the Acom, and the proximal A2 segment (Fig. 1A, B). The Acom/A2 angle was measured on the plane containing the distal portion of the ipsilateral A1 segment. The view measuring the Acom/A2 angle was made by rotating 90° around the center axis of the distal A1 segment from the view measuring the A1/Acom-A2 plane angle. Two investigators independently performed the angle measurement. First, the initial analysis was performed using the data from the first observer (K.S.). Then, the reproducibility of the results was evaluated using the data from the second observer (A.O.). The diameters and angles were measured using a three-dimensional image processing workstation, the Ziostation2 (Ziosoft Inc., Tokyo, Japan).

### Statistics

Inter-observer agreement for the presence or absence of the growth of Acom aneurysms was assessed by overall percent agreement and the unweighted κ statistics. Continuous variables were evaluated with the Wilcoxon rank-sum test. Categorical variables were compared with Pearson χ^2^ test or Fisher’s exact test as appropriate. Non-parametric multiple comparisons were conducted using the Steel-Dwass test. Univariate and multivariate logistic regression models were applied to evaluate the association of each variable with progression. A *P*-value less than 0.10 on univariate analysis was used as the cut-off value for the multivariate logistic regression model. Odds ratios (ORs) with 95% confidence intervals (CIs) were calculated, and a *P*-value smaller than 0.05 was defined as significant. To investigate the correlation between the largest dimension of Acom aneurysms and the Acom-A2 bifurcation angles, scatter diagrams with a linear regression line were prepared, and Spearman’s correlation analysis was performed.

It was thought that the results of the association between the bifurcation angle and progression in the case–control study might be affected by very small or large aneurysms due to a potential correlation between the bifurcation angle and the size of sIAs. Therefore, a sensitivity analysis was performed to assess factors associated with progression using subgroup data acquired from patients with Acom aneurysms that were 3–7 mm in the largest dimension. All statistical analyses were performed with EZR version 1.54 (Saitama Medical Center, Jichi Medical University, Saitama, Japan)^23^.

## Results

### Types of saccular aneurysms in the rat aneurysm model

**Figure 2:**
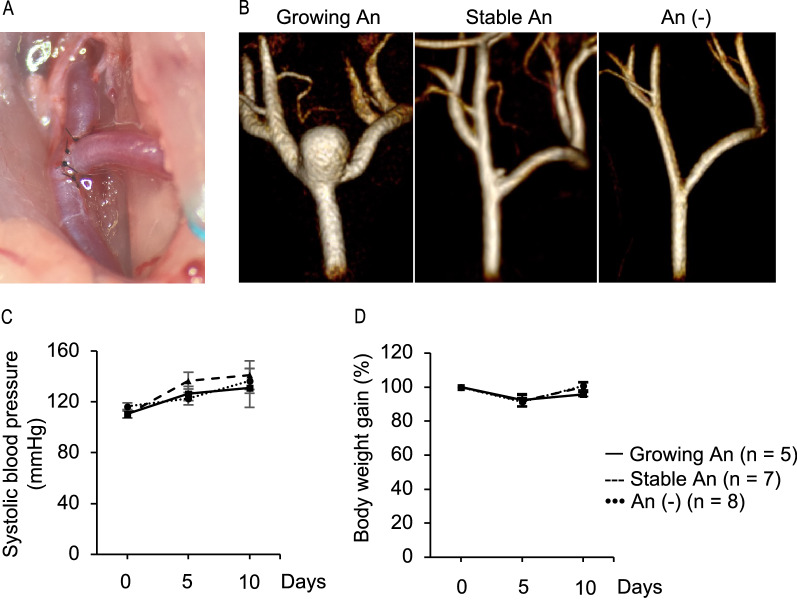
Present saccular aneurysm model at the surgically created common carotid artery (CCA) bifurcation in rats. (**A**) A macroscopic view of the surgically created CCA bifurcation. (**B**) Representative magnetic resonance angiography findings of the CCA bifurcation one month after aneurysm induction demonstrating the three categories: a growing aneurysm (An) (the left panel), a stable An (the middle panel), and no An (the right panel). These images were created using Horos visualization software (64-bit, version 3.6.6, https://horosproject.org). Systolic blood pressure (**C**) and body weight (**D**) before and on the 5th and 10th days after aneurysm induction. Data are presented as means ± SD. Statistical comparisons were performed by the Steel-Dwass test at each time point.

Saccular aneurysms were induced at the surgically created bifurcation site shown in Fig. 2A in 45 rats. Induced aneurysms were categorized into three types based on the aneurysm size one month after the surgical manipulations: growing aneurysms ≥ 1 mm in the largest dimension, stable aneurysms < 1 mm in the largest dimension, and no aneurysms at the CCA bifurcation (Fig. 2B). MRI examinations on the 30th day postoperatively were performed in all the rats that survived. Histological specimens were used if MRI examinations could not be performed because of sudden death attributable to rupture of lesions. The frequency of each category was 44.4% (20/45) for growing aneurysms, 33.3% (15/45) for stable aneurysms, and 22.2% (10/45) for no aneurysms. Among the 45 rats, three were categorized only based on histological specimens because of aneurysm rupture, all of which were classified to growing aneurysms.

Blood pressure and body weight were assessed after the surgical manipulations in 20 animals. Systolic blood pressure and body weight during the first ten days after the surgical manipulations were similar among the three categories (Fig. 2C, D).

### Bifurcation angle predicts the progression of saccular aneurysms in the rat model

**Figure 3:**
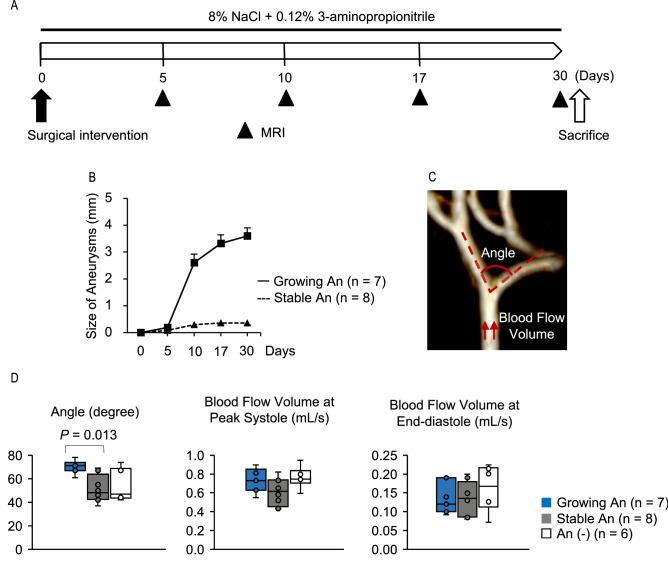
The association of the bifurcation angle with aneurysm progression in rats. (**A**) A schematic representation of the study design. Magnetic resonance imaging (MRI) includes MR angiography and cardiac-gated two-dimensional phase-contrast MRI. (**B**) Sequential assessments by MR angiography showing the size of growing (n = 7) and stable (n = 8) aneurysms (An). Data are presented as means ± SD. (**C**) MR angiography showing the bifurcation angle and the point where blood flow volume estimated by phase-contrast MR imaging was measured. This image was created using Horos visualization software (64-bit, version 3.6.6, https://horosproject.org). (**D**) Comparison of the bifurcation angle (the left panel) and blood flow volume at peak systole (the middle panel) and end-diastole (the right panel) between growing (n = 7), stable (n = 8), and no An (n = 6) on the 5th day after the aneurysm induction. Data are shown as box-and-whisker plots. Statistical comparisons were performed by the Steel-Dwass test.

Sequential morphological assessments of induced aneurysms were performed by MRI in 21 of 45 rats. MRI was performed at 5, 10, 17, and 30 days after the surgical manipulations (Fig. 3A). Of the 21 animals, seven had growing aneurysms, eight had stable ones, and six had no aneurysms. Three of the seven growing aneurysms ruptured between the 11th and 30th days postoperatively.

Because the bifurcation angles might become wider as induced aneurysms grew, we first determined the time point that could be used as a control for evaluating the association of the bifurcation angle with subsequent growth. The size of growing aneurysms was similar to that of the stable ones until the 5th day and then significantly increased between the 5th and 10th days after the surgical manipulations (Fig. 3B). Thus, the bifurcation angle and blood flow volume through the proximal CCA were evaluated on the 5th day as the baselines before the progression (Fig. 3C). On the 5th day, the bifurcation angles were significantly wider in the growing aneurysms (median, 71°; interquartile range (IQR), 69°–73°) compared to those in the stable ones (median, 48°; IQR, 43°–58°) (*P* = 0.013), whereas blood flow volume at the proximal CCA was similar between the groups (Fig. 3D). The box-and-whisker plots showed a threshold of 61° in the bifurcation angle for the growing aneurysms (Fig. 3D). The bifurcation angles in the no aneurysm group (median, 47°; IQR, 45°–62°) were similar to those in the stable group (Fig. 3D).

### Clinical and radiological factors associated with the progression of Acom aneurysms

**Figure 4:**
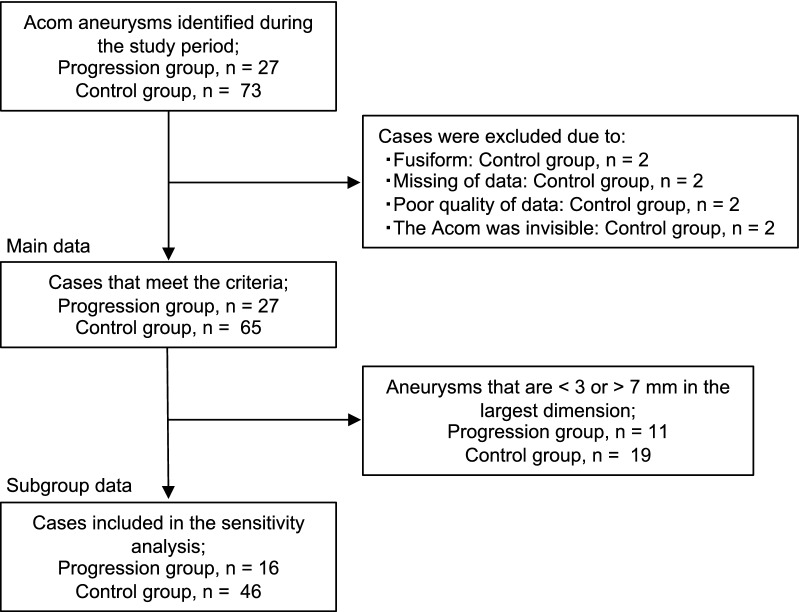
Patient enrollment flowchart in the case–control study. *Acom* anterior communicating artery.

Eight patients in the control group were excluded from the analysis because of the following reasons: aneurysms were fusiform in two patients, radiological data at the time of diagnosis were unavailable or poor in four, and the Acom could not be visualized in two. In total, 27 patients in the progression group and 65 in the control group were included in the analysis (Fig. 4). Inter-observer agreement for the presence or absence of the growth was excellent; the overall percent agreement was 92.4% (85/92), and κ coefficient was 0.82 (95% CI, 0.61–1.0). The median period from diagnosis to detection of progression was 48 (IQR, 20–70) months. Six cases (22.2%) ruptured, and all these aneurysms had grown when rupture. The largest dimension after progression among the 27 cases was a median of 5.7 (IQR, 4.9–7.4) mm. The median difference in the largest dimension between the time of diagnosis and that of progression was 1.7 (IQR, 1.3–2.7) mm. In the control group, the median follow-up period was 47 (IQR, 37–60) months.

Baseline patient and radiological characteristics are presented in Table 1. On univariate analyses, female sex, history of dyslipidemia, and the Acom/A2 angle were associated with progression. After adjustment by the logistic regression model, female sex (OR, 4.79; 95% CI, 1.59–14.5; *P* = 0.005), history of dyslipidemia (OR, 3.31; 95% CI, 1.16–9.47; *P* = 0.026), and wider Acom/A2 angle (OR, 1.01; 95% CI, 1.00–1.03; *P* = 0.035) were significantly associated with progression. In the Acom/A2 angle, the ORs for the progression of the second (145°–179°; progression group, n = 12; control group, n = 19) or third (180°–274°; progression group, n = 12; control group, n = 19) tertiles compared to the first tertile (46°–143°; progression group, n = 3; control group, n = 27) were 5.5 (95% CI, 1.3–35).

**Table 1:** Baseline patient and radiological characteristics of anterior communicating artery aneurysms with (n = 27) or without (n = 65) progression.

Characteristics	Progression group (n = 27)*	Control group (n = 65)*	Univariate analysis			Multivariate analysis		
			OR	95% CI	*P* value	OR	95% CI	*P* value
**Patient characteristics**								
Age, y (IQR)	66 (60–74)	62 (53–71)	0.96	0.93–1.01	0.16			
Female, n (%)	20 (74)	28 (43)	3.72	1.29–11.95	0.01	4.79	1.59–14.5	0.005
Hypertension, n (%)	17 (63)	32 (49)	1.74	0.64–4.95	0.23			
Dyslipidemia, n (%)	14 (52)	18 (28)	2.78	1.00–7.88	0.048	3.31	1.16–9.47	0.026
Diabetes mellitus, n (%)	2 (7)	9 (14)	0.5	0.05–2.68	0.50			
Smoking, n (%)	7 (26)	23 (35)	0.64	0.20–1.89	0.52			
**Radiological characteristics**								
Largest dimension, mm (IQR)	3.8 (2.6–5.0)	3.7 (3.0–5.5)	0.93	0.72–1.20	0.67			
Neck size, mm (IQR)	3.1 (2.2–4.3)	3.0 (2.2–4.0)	1.01	0.69–1.47	0.77			
Acom/A2 angle, degrees (IQR)	179 (155–192)	149 (120–187)	1.02	1.00–1.03	0.01	1.01	1.00–1.03	0.035
A1/Acom-A2 plane angle, degrees (IQR)	42 (12–59)	30 (10–66)	1.00	0.99–1.01	0.95			
A1 diameter, mm (IQR)	2.4 (2.0–2.6)	2.4 (2.0–2.5)	0.65	0.22–1.98	0.65			
A2 diameter, mm (IQR)	1.7 (1.5–2.1)	1.9 (1.6–2.2)	0.56	0.18–1.79	0.35			
Acom diameter, mm (IQR)	1.7 (1.4–1.9)	1.6 (1.3–2.0)	0.90	0.35–2.30	0.99			
Contralateral A1 diameter, mm (IQR)	0.9 (0–1.2)	1.1 (0–1.6)	0.66	0.36–1.22	0.17			

*Data are shown as n (%) or median (IQ R) values.

Statistical analyses included in this table were performed using EZR version 1.54^23^.

*CI* confidence interval, *IQR* interquartile range, *OR* odds ratio.

Inter-observer variation of the Acom/A2 bifurcation angle was 6° (IQR, 4°–9°). The multivariate logistic regression analysis using the data from the second observer reproduced the significant association of the Acom/A2 bifurcation angle with the progression (Supplementary Table 1). Besides, the sensitivity analysis also confirmed the significant associations of female sex, history of dyslipidemia, and wider Acom/A2 angle with progression (Supplementary Table 2).

### Correlation analysis between the bifurcation angle and the size of Acom aneurysms

**Figure 5:**
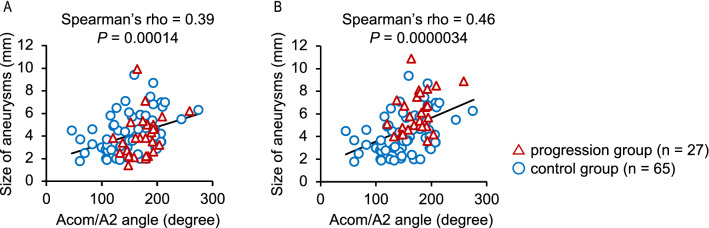
Scatter diagrams with a linear regression line showing the correlation between the largest dimension of anterior communicating artery (Acom) aneurysms and the angle formed between the Acom and the A2 segment of the ACA (Acom/A2 angle). Spearman’s correlation analysis was performed at each time point before (**A**) and after (**B**) progression.

In order to assess the potential contribution of the Acom/A2 angle to the growth of Acom aneurysms, the correlation between the Acom/A2 angle and the largest dimension of aneurysms was evaluated in all cases. The correlation was assessed using the data of the largest dimension at two different time points for the progression group, i.e., before (Fig. 5A) and after (Fig. 5B) the progression of the aneurysms. Scatter diagrams demonstrated a significant positive linear correlation between the Acom/A2 angle and the largest dimension of the aneurysms both before (Spearman’s rho, 0.39; *P* = 0.00014) and after (Spearman’s rho, 0.46; *P* = 0.0000034) progression. In addition, the scatter diagrams showed an Acom/A2 angle threshold of 120° for progression.

## Discussion

The present study using a rat model, where factors such as body weight, blood pressure, blood flow volume at the inflow, and sex were aligned, confirmed that the bifurcation angle was significantly associated with the progression of saccular aneurysms. In the case–control study of Acom aneurysms, the clinical relevance of the bifurcation angle to the progression of sIAs was demonstrated; aneurysm progression was more frequently observed in the cases with the bifurcation angle ≥ 145° than in the remaining cases, with the OR of 5.5 (95% CI, 1.3–35). The correlation analysis between the Acom-A2 bifurcation angles and the largest dimension of Acom aneurysms showed a positive correlation, indicating a causal relationship between the two parameters. In these analyses, thresholds in bifurcation angles were observed, i.e., aneurysm progression occurred only in cases where bifurcation angles were ≥ 120° in Acom aneurysms and ≥ 61° in the rat model. These findings suggest that the bifurcation angle may be a predictor of progression.

Past cross-sectional studies comparing the bifurcation angles with sIAs and those without sIAs consistently showed that wider angles were associated with the presence of lesions^8–10,12,24^. The bifurcation angle is, therefore, likely to contribute to aneurysm formation. Meanwhile, the significance of the bifurcation angle in the progression of already-formed sIAs had not been clarified. To the best of our knowledge, this is the first longitudinal study demonstrating that wider bifurcation angles are associated with the progression of sIAs. In this regard, Rashad et al. reported that bifurcation angles were smaller in ruptured sIAs than in unruptured ones^25^; however, this study should not be regarded as demonstrating the association of the bifurcation angle with the progression of already-formed sIAs because of its nature as a cross-sectional study^25^. Nevertheless, this study was consistent with the present study in terms of the positive association of aneurysm size with bifurcation angles^25^. These findings imply that the bifurcation angle should be adjusted by aneurysm size when considered as a predictor of progression.

We defined aneurysm progression as growth or rupture during observation. Considering that all cases that ruptured during the follow-up period showed growth before rupture, the findings in the present study suggest that the bifurcation angle was associated with the growth of sIAs. Meanwhile, whether growth is considered to be a surrogate marker for rupture has been carefully assessed. Past studies have demonstrated that the rupture rates of sIAs showing growth were significantly higher than of sIAs without growth (3.1% per year vs. 0.1% per year, *P* < 0.01)^15^. In addition, according to meta-analyses, most risk factors for growth, such as female sex, older age, size, and irregular shape, are consistent with those for rupture^14,15,26,27^. Furthermore, recent studies have suggested that inflammation visualized by macrophage imaging or gadolinium-enhanced MRI may predict both growth and rupture^28–33^. These findings combined suggest that growth and rupture share similar pathogenetic mechanisms, indicating that growth is a useful surrogate marker for rupture.

Of the patient characteristics analyzed in the present study, female sex and history of dyslipidemia were associated with the progression of sIAs. The association of female sex with the growth or rupture of sIAs was consistent with a previous meta-analysis^15,26^. This may be attributable to postmenopausal estrogen deficiency, because similar findings have been reproduced by ovariectomy in female rodent models^34,35^. As for dyslipidemia, the association with the progression of sIAs has been controversial. Recently, several studies have reported that dyslipidemia increased the risk of sIA rupture^36,37^. This is supported by a previous study showing that a high-fat diet intake promoted the growth of sIAs in a rodent model^38^. Meanwhile, statins, which are frequently used in patients with dyslipidemia, have been shown to lower the risk of rupture^39,40^. Therefore, the involvement of dyslipidemia in the progression of sIAs could have been masked by statin use. Further study is required to settle this controversy.

There are several limitations in the present study. First, retrospectively collected data in a 1:2 ratio between cases and controls were analyzed in the human study. Therefore, prospective studies are needed to estimate the hazard ratio of the bifurcation angle in predicting sIA progression. Second, considering the period from diagnosis to progression in Acom aneurysms, the follow-up period for some patients might not be long enough to detect potential growth. Third, there was a subgroup of patients unsuitable to the angle measurement by the present methods because of the poor quality of radiological examinations or the morphology of the Acom artery itself; therefore, the present methods cannot be applied in such patients. Fourth, the significance of the bifurcation angle needs to be verified in other bifurcation sites, because the optimal angle determined by the radii and blood flow volume depends on anatomical location^3,4^. Fifth, the mechanism underlying the potential contribution of the bifurcation angle to aneurysm progression has not been determined. Thus, we could not answer why the majority of cases with wide Acom/A2 bifurcation angles remained stable. Because the pathogenesis of sIAs is mediated by macrophage-mediated inflammatory responses, widening of the bifurcation angle presumably triggers inflammation through mechanosensing of hemodynamic stress loaded there^41,42^. Finally, a rat CCA aneurysm model was used. However, differences in wall structure between intracranial and extracranial arteries should be acknowledged.

## Conclusions

The present animal study results suggest that the bifurcation angle is positively associated with the progression of saccular aneurysms. The case–control study of Acom aneurysms demonstrates the reproducibility of the findings acquired from the animal study in humans. The OR for the progression in Acom aneurysms with the bifurcation angle ≥ 145° (i.e., the second or third tertiles) compared to those with the bifurcation angle less than 145° was 5.5. In addition, the largest dimensions of Acom aneurysms were positively correlated with bifurcation angles, indicating a potential contribution of the bifurcation angle to aneurysm growth. Furthermore, these analyses indicate the presence of thresholds of bifurcation angles for aneurysm progression. These findings imply the usefulness of the bifurcation angle for predicting the progression of sIAs.

## Supplementary Information

Supplementary Tables.

## Abbreviations

Acom: Anterior communicating artery
CCA: Common carotid artery
CI: Confidence interval
IQR: Interquartile range
MRI: Magnetic resonance imaging
OR: Odds ratio
sIAs: Saccular intracranial aneurysms
