# Supplementary material for: The bifurcation angle is associated with the progression of saccular aneurysms
Source: Sci Rep. 2022 May 6;12:7409. doi: 10.1038/s41598-022-11160-5 (PMC9076676; doi:10.1038/s41598-022-11160-5)
Supplement: Supplementary file 1 — Supplementary Tables. [file 41598_2022_11160_MOESM1_ESM.pdf]

The bifurcation angle is associated with the progression of saccular aneurysms

Running title: Bifurcation angle predicts unstable aneurysms

Kampei Shimizu[1, 2], Hiroharu Kataoka[1, 3, *], Hirohiko Imai[4], Takeshi Miyata[1], Akihiro Okada[1, 2], Nobuyuki Sakai[5], Masaki Chin[6], Koichi Iwasaki[7], Taketo Hatano[8], Hirotoshi Imamura[5], Ryota Ishibasi[6], Masanori Goto[7], Masaomi Koyanagi[8], Tomohiro Aoki[2], and Susumu Miyamoto[1]

[1]Department of Neurosurgery, Kyoto University Graduate School of Medicine, Kyoto, 606-8507, Japan

[2]Department of Molecular Pharmacology, Research Institute, National Cerebral and Cardiovascular Center, Suita, 564-8565, Japan

[3]Department of Neurosurgery, National Cerebral and Cardiovascular Center, Suita, 564-8565, Japan

[4]Department of Systems Science, Graduate School of Informatics, Kyoto University, Kyoto, 606-8507, Japan

[5]Department of Neurosurgery, Kobe City Medical Center General Hospital, Kobe, 650-0047, Japan

[6]Department of Neurosurgery, Kurashiki Central Hospital, Kurashiki, 710-8602, Japan

[7]Department of Neurosurgery, Tazuke Kofukai Medical Research Institute and Kitano Hospital, Osaka, 530-8480, Japan

[8]Department of Neurosurgery, Kokura Memorial Hospital, Kokura, 802-8555, Japan

Supplementary Table 1. Multivariate logistic regression analysis by the second observer for assessing the bifurcation angle in anterior communicating artery aneurysms with (n = 27) or without (n = 65) progression

Characteristics	Progression group (n=27)*	Control group (n=65)*	Univariate analysis			Multivariate analysis		
			OR	95% CI	*P* value	OR	95% CI	*P* value
Patient characteristics								
Female, n (%)	20 (74)	28 (43)	3.72	1.29-11.95	0.01	4.81	1.59-14.6	0.003
Dyslipidemia, n (%)	14 (52)	18 (28)	2.78	1.00-7.88	0.048	3.44	1.20-9.88	0.019
Radiological characteristics								
Acom/A2 angle, degrees (IQR)	177 (157-188)	151 (116-185)	1.02	1.00-1.03	0.006	1.02	1.00-1.03	0.014

*Data are shown as n (%) or median (IQR) values. Statistical analyses included in this table were performed using EZR version 1.54.[1]
Abbreviations: CI = confidence interval, IQR = interquartile range, OR = odds ratio

Supplementary Table 2. Sensitivity analysis in patients with anterior communicating artery aneurysms that are 3-7 mm in the largest dimension

Characteristics	Progression group (n=16)*	Control group (n=46)*	Univariate analysis			Multivariate analysis		
			OR	95% CI	*P* value	OR	95% CI	*P* value
Patient characteristics								
Age, yrs (IQR)	62 (55-71)	67 (62-74)	0.93	0.86-1.00	0.078	0.72	0.56-0.92	0.0081
Women, n (%)	12 (75)	17 (37)	4.98	1.25-24.64	0.019	205	4.9-8590	0.0053
Hypertension, n (%)	11 (69)	25 (54)	1.83	0.49-7.84	0.48			
Dyslipidemia, n (%)	9 (56)	13 (28)	3.2	0.86-12.55	0.087	295	4.6-18900	0.0073
Diabetes mellitus, n (%)	2 (13)	6 (13)	0.95	0.09-6.19	1			
Smoking, n (%)	6 (38)	17 (37)	1.02	0.26-3.80	1			
Radiological characteristics								
Largest dimension, mm (IQR)	4.5 (3.8-5.1)	4.5 (3.5-5.7)	0.92	0.57-1.49	0.95			
Neck size, mm (IQR)	3.7 (3.1-4.5)	3.3 (2.7-4.1)	1.36	0.78-2.37	0.22			
Acom/A2 angle, degree (IQR)	185 (167-193)	153 (127-192)	1.01	1.00-1.03	0.0497	1.04	1.01-1.08	0.019
A1/Acom-A2 plane angle, degree (IQR)	26 (4-62)	32 (10-67)	0.99	0.98-1.01	0.50			
A1 diameter, mm (IQR)	2.4 (2.0-2.5)	2.4 (2.1-2.6)	0.49	0.12-2.07	0.37			
A2 diameter, mm (IQR)	1.7 (1.5-2.0)	1.9 (1.6-2.3)	0.38	0.09-1.59	0.16			
Acom diameter, mm (IQR)	1.8 (1.3-1.9)	1.7 (1.4-2.1)	0.74	0.24-2.29	0.73			
Contralateral A1 diameter, mm (IQR)	0.5 (0-1.1)	1.3 (0-1.6)	0.46	0.21-1.04	0.0497	0.11	0.02-0.60	0.011

*Data are shown as n (%) or median (IQR). Statistical analyses included in this table were performed using EZR version 1.54.[1]
Abbreviations; CI = confidence interval, IQR = interquartile range, OR = odds ratio

References

1. Kanda, Y. Investigation of the freely available easy-to-use software 'EZR' for medical statistics. *Bone Marrow Transplant.* **48**, 452–8 (2013).
